# Supplementary material for: Recent Advances in Nanomaterial-Based Biosensors for Pesticide Detection in Foods
Source: Biosensors (Basel). 2022 Jul 27;12(8):572. doi: 10.3390/bios12080572 (PMC9405907; doi:10.3390/bios12080572)
Supplement: Supplementary file 1 [file biosensors-12-00572-s001.zip › biosensors-1794260-SUPPLEMENTARY MATERIAL- 4th proofeading.pdf]

SUPPLEMENTARY MATERIAL

Recent advances in nanomaterial-based biosensors for pesticide detection in foods

Ana Carolina de Morais Mirres [1,2,3,4,5,†], Brenno Enrique Pereira de Matos da Silva [1,2,3,4,5,†], Leticia Tessaro [2,3,4,5,6], Diego Galvan [2,3,4,5,6], Jelmir Craveiro de Andrade [2,3,4,5,6], Adriano Aquino [2,3,4,5,6], Nirav Joshi [2,3,4,5] , and Carlos Adam Conte-Junior [2,3,4,5,6,*]

Corresponding author. Departamento de Bioquímica, Instituto de Química, Universidade Federal do Rio de Janeiro (UFRJ), Rio de Janeiro, RJ, 21941-901, Brasil. conte@iq.ufrj.br (Conte-Junior C.A.).

Table S1 presents all LMR values established by the Codex Alimentarius [1] and European Commission [2] the respective pesticides applied.

LOD	Pesticide	Food matrix	LMR ($mg\ kg^{-1}$)		Ref
			CODEX	E.U.	
**2,17 x10^{-2} ngL^{-1}**	**MP**	**Chinese cabbage**	0.05	0.01	[3]
19 - 77 ngL^{-1}	11 pesticides OPPs	Apple<br>cabbage	-	-	[4]
81 ng L^{-1}	methomyl	Apple<br>cabbage	0.3<br>NAC	0.01<br>0.01	
1.0 nM	Carbamate	Fruit	-	-	[5]
CPF : 36 ng L^{-1}	Chlorpyrifos	Apple<br>Pak choi	NAC<br>NAC	0.01<br>NAC	[6]
70 x10^{-3} ng L^{-1}	Chlorpyrifos	Chinese cabbage<br>Lettuce	1.0<br>NAC	0.01<br>0.01	[7]
2.48x10^{3} ng L^{-1}	Isocarbophos	Cabbage<br>Peach<br>Tea	NAC<br>NAC<br>NAC	NAC<br>NAC<br>NAC	[8]
7.12 x 10^{-5} nM	Acetamiprid	Tea	NAC	0.05	[9]
1x 10 $^{-6}$ nM	Malathion	Lettuce	NAC	0.5	[10]
17.18 ng L^{-1}	Malathion	Cucumber<br>long bean	0.2<br>1.0	0.02<br>0.02	[11]
17 ng L^{-1}.	MP	Apple<br>Carrot	0.2<br>NAC	0.01<br>0.01	[12]
34 ng L^{-1}	OPs	Apple	-	-	[13]
4x10^{3} ng L^{-1}	Paraoxon	Chinese chives<br>Cabbage	NAC<br>NAC	0.02<br>0.01	[14]
5 x 10 $^{-4}$ nM	Malathion	Apple	0.5	0.02	[15]
212 nM	Paraoxon	Milk	NAC	0.01	[16]
8.2 nmol L^{-1}	MP	Apple<br>Cabbage,<br>Spinach<br>Lettuce	0.2<br>0.05<br>NAC<br>NAC	0.01<br>0.01<br>0.01<br>0.01	[17]
Fenthion:1.3 nM<br>Dichlovos: 5.3 nM	Fenthion<br>Dichlorvos	Cabbage juice	NAC	0.01	[18]
0.36 nM	Acetamiprid	Celery leaves<br>Chinese green tea	NAC	NAC	[19]
Methyl parathion:3,04x10^{-3} ng L^{-1}<br>Malathion:1,96x10^{-3} ng L^{-1}<br>chloropyrifos: 2,060x10^{-3} ng L^{-1}	OPs	Cabbage water	-	-	[20]
NC : 2.40 x 10 $^{-3}$ nM	ethyl parathion	Orange and Apple juice	NAC	NAC	[21]
MHCS: 14.8 ng L-1<br>Fe3O4@MHCS: 18.2 ng L^{-1}	Malathion	Pear	1.0	0.02	[22]
8.6x10^{-6} - 7.1 x10^{-5} nM	Chitosan	Potato<br>Corn grans	NAC<br>NAC	NAC<br>NAC	[23]
	malathion	Potato<br>Corn grans	NAC<br>0.02	0.02<br>8.0	
	MP	Potato<br>Corn grans	0.05<br>NAC	0.01<br>0.02	
	chlorpyrifos	Potato<br>Corn grans	2.0<br>0.01	0.01<br>0.01	
Chlorpyrifos 730 ng L^{-1}	Chlorpyrifos	Maize	0.05	0.01	[100]

diazinon 6.7x10 ^{3} ng L^{-1} malathion 740 ng L^{-1} ,		long bean	0.01	0.01	
		cauliflower	0.05	0.01	
		eggplant	NAC	0.01	
		oyster mushroom	NAC	NAC	
		shiitake mushroom	NAC	NAC	
		apple	NAC	0.01	
		orange	NAC	0.01	
		tomato	NAC	0.01	
		blueberry	NAC	0.01	
		spinach	NAC	0.01	
		lettuce	NAC	0.01	
		cabbage	1.0	0.01	
	Malathion	Maize	0.05	8.0	
		long bean	1.0	NAC	
		cauliflower	NAC	0.02	
		eggplant	NAC	0.02	
		oyster mushroom	NAC	NAC	
		shiitake mushroom	NAC	NAC	
		apple	0.5	0.02	
		orange	NAC	2.0	
		tomato	0.5	0.02	
		blueberry	NAC	NAC	
		spinach	3.0	0.02	
		lettuce	NAC	0.5	
		cabbage	NAC	0.02	
	Diazinon	Maize	0.02	0.01	
		long bean	0.2	0.01	
		cauliflower	NAC	0.01	
		eggplant	NAC	0.01	
		oyster mushroom	NAC	NAC	
		shiitake mushroom	NAC	NAC	
		apple	NAC	0.01	
		orange	NAC	0.01	
		tomato	0.5	0.01	
		blueberry	NAC	NAC	
		spinach	0.5	0.01	
		lettuce	0.5	0.01	
		cabbage	0.5	0.01	
2 ng L^{-1}	Parathion	pear	0.2	0.05	[24]
		cabbage	NAC	0.05	
		rice	NAC	0.05	
2.13 ng kg^{-1}	Parathion	rice	NAC	0.05	[24]
		pear	0.2	0.05	
		apple	0.2	0.05	
		cabbage	NAC	0.05	
50 nM	DMMP	apple juice	US	US	[25]
5×10^{-1} ng L^{-1}	Malathion	cauliflower	NAC	0.02	[26]
		cabbage	NAC	0.02	
1.4x 10 ^{3} ng L^{-1}	Paraoxon	Vegetable (not specified)	US	US	[27]
50 ng L^{-1}.	Chlorpyrifos	cabbage	1.0	0.01	[28]
		rape	NAC	NAC	
		lettuce	NAC	0.01	
0.3 nmol L^{-1}	Diazinon	lettuce juice	0.5	0.01	[29]
		tomato juice	0.5	0.01	
1x10^{-6} nM	Malathion	lettuce	NAC	0.5	[30]
0.1 nM	Paraoxon	potato	US	US	[31]
0.004 nM	Organophosphate pesticide	spinach and cabbage	-	-	[32]

1 ng L^{-1}	Chlorpyrifos-methyl	lettuce	NAC	0.01	[33]
0.7nM	Acetamiprid	cabbage leaves	0.7	0.4	[34]
3x10^{-6} nmol L^{-1}	Paraoxon	spinach juice	US	US	[35]
Malathion : 3.11 x 10^{-4} ng L^{-1} metyl parathion : 1.88 x 10^{-4} ng L^{-1}	Malathion	chinese cabbage	NAC	0.02	[58]
	Methyl parathion	chinese cabbage	0.05	0.01	
20 ng L^{-1}	Chlorpyrifos	cabagge	NAC	0.01	[37]
		spinach	NAC	0.01	
0.23 nM	Dichlorvos	cabagge juice	NAC	0.01	[38]
methyl paraoxon: 240 nM methyl parathion: 260 nM ethyl paraoxon: 220 nM	OPs	Lactuca sativa L	-	-	[61]
1.3 x10^{3} ng L^{-1}	Profenofos	Chinese chives	NAC	0.05	[62]
Monocrotophos 2510 ng L^{-1} Dimethoate 1500 ng L^{-1}	Monocrotophos Dimethoate	Soft drinks	-	-	[40]
3.6 nM	Phosmet	Wheat flour	NAC	NAC	[41]
0.2 nM	Pirimiphosmethyl	Olive oil	US	US	[42]
Paraoxon 1.62 x10^{-6} nM Dichlorvos 7.53 x10^{-5} nM Malathion 0.23 nM Triazophos 1.06 x10^{-2} nM .	Paraoxon	apple juice	US	US	[43]
		tomato juice	US	US	
	Dichlorvos	apple juice	NAC	0.01	
		tomato juice	NAC	0.01	
	Malathion	apple juice	0.5	0.02	
		tomato juice	0.01	0.02	
	Triazophos	apple juice	NAC	0.01	
		tomato juice	NAC	0.01	
1ng L^{-1}	Malathion	Lettuce leaves	NAC	0.5	[44]
1.9 nmol L^{-1}	Carbamate pesticide	Tomatoes	-	-	[45]
1.70×10^{3} ng/L	Paraoxon	apple and eggplant	-	-	[12]
3.9x10^{2} ng L^{-1}	Malathion	cabbage	NAC	0.02	[46]
		carrots	NAC	0.02	
	Organophosphate inseticide	cabbage	-	-	
		carrots			
1.7 x10^{-3} nM	Organophosphate pesticide	Cabbage and Cucumber	-	-	[47]
Pirimicarb: 5 x10^{4} ng L^{-1} dichlorvos: 1 x10^{4} ng L^{-1} carbaryl: 1 x10^{4} ng L^{-1}	Organophosphorus and carbamate insecticides	Lettuce, choy, and rice samples	-	-	[48]
biosensors: 26 nM	Nitrofen	Apricot	US	0.01	[49]
acetochlor : 6.3 x10^{2} ng L^{-1} fenpropathrin:2.4 x10^{2} ng L^{-1}	Acetochlor	Corn	0.04	0.01	[50]
		sorghum	NAC	0.01	
		soybean	UC	UC	
		apple	NAC	0.01	
		orange	NAC	0.01	
		peach	NAC	0.01	
		cabbage	NAC	0.01	
		broccoli	NAC	0.01	
		tomato	NAC	0.01	
		drinking water	NAC		
	Fenpropathrin	Corn	NAC	0.01	
		sorghum	NAC	0.01	
		soybean	UC	UC	
		apple	NAC	0.01	
		orange	NAC	2.0	
		peach	NAC	0.01	
		cabbage	NAC	0.01	
		broccoli	NAC	0.01	
		tomato	1.0	0.01	
		drinking water	UC	UC	
1.67 × 10^{-3} nM	OPs	Pakchoi, cabbage and lettuce	-	-	[51]

50 ng·L^{-1}	Carbendazim	Apple<br>cucumber<br>matcha powder	NAC<br>0.05<br>UC	0.2<br>0.1<br>UC	[52]
29 nM	Dichlorvos	cabbage juice	NAC	0.01	[53]
70 ng L^{-1}	Chlorpyrifos	Spiked apple	NAC	0.01	[54]
		celery cabbage	1.0	0.01	
